# Carfilzomib-induced thrombotic microangiopathy (TMA): an under-recognised spectrum of disease from microangiopathic haemolysis to subclinical TMA

**DOI:** 10.1038/s41408-023-00885-9

**Published:** 2023-07-26

**Authors:** Royston Ponraj, Adam Bryant, Lindsay Dunlop, Heather Range, Cherry Cobrador, Silvia Ling, Danny Hsu

**Affiliations:** 1 Haematology Department, Liverpool Hospital, Sydney, Australia; 2 School of Medicine, University of New South Wales, Sydney, Australia; 3 School of Medicine, Western Sydney University, Sydney, Australia; 4 Department of Pharmacy, Liverpool Hospital, Sydney, Australia

**Keywords:** Chemotherapy, Myeloma, Myeloma

Dear Editor,

Carfilzomib is an effective therapy for multiple myeloma. However, carfilzomib-induced thrombotic microangiopathy (TMA) has emerged as a potential severe complication, characterised by microangiopathic haemolytic anaemia (MAHA) together with end organ damage, particularly kidney injury [1, 2].

Carfilzomib is an irreversible inhibitor of the ubiquitin-proteasome pathway, thought to cause complement dysregulation [1, 3]. Excessive complement activation leads to endothelial injury resulting in microvascular thrombi, consumptive thrombocytopenia, haemolytic anaemia and end organ damage [4, 5]. Carfilzomib also results in the reduction of vascular endothelial growth factor (VEGF) levels via NFκB inhibition, which is known to cause glomerular endothelial injury and renal TMA [1, 6, 7]. In combination with these drug effects, certain patient factors, such as inherent complement gene variants, may predispose individuals to TMA with carfilzomib exposure [2, 8].

In carfilzomib-treated patients, we observed episodes of haemolysis associated with peripheral schistocytes in patients without overt TMA clinical features. We hence evaluated the incidence and characteristics of MAHA associated with carfilzomib at our centre, demonstrating a spectrum between low-grade MAHA, associated acute kidney injury and severe TMA.

A retrospective study identified 46 patients with multiple myeloma treated with carfilzomib between January 2010 and December 2019 at a single tertiary centre. Given the median onset of carfilzomib-induced TMA is reported to be 3 months, to reduce bias, we excluded patients that had received two or less cycles [6]. Therefore, of the total 46 patients identified, 14 were excluded. All 14 patients ceased carfilzomib either due to disease progression or death related to multiple myeloma. One patient was excluded due to incomplete records.

For the remaining 31 suitable patients, we collected pathology data at multiple time points, including the two highest lactate dehydrogenase (LDH) peaks during therapy. We defined strict criteria for sufficient evidence of MAHA as: schistocytes on a peripheral blood film, together with anaemia and thrombocytopenia with a fall in platelet count of >25% from baseline, as well as one or more positive markers of haemolysis. Elevated LDH was required. Other haemolytic markers could include elevated bilirubin, reticulocytosis, reduced haptoglobin or the presence of urinary haemosiderin. TMA was defined as MAHA together with end organ dysfunction [4]. Acute kidney injury (AKI) was defined according to KDIGO guidelines as an increase in serum creatinine by >26.5 µmol/L, or >50% of baseline over 7 days [9].

In addition to the above criteria, all data time points were correlated with relevant multiple myeloma disease marker (M protein) to account for potential confounders. Hence, episodes of LDH elevation due to tumour lysis within the first cycle were excluded. Similarly, cytopenias and renal failure due to progressive myeloma were excluded.

Amongst the 31 patients, the median age was 67 years (range 51–84). This was a culturally diverse population with six ethnic groups represented. All patients had relapsed multiple myeloma, with a median of 3 previous treatment lines. Almost all patients were treated with carfilzomib 56 mg/m^2^ twice weekly in combination with dexamethasone for 3 weeks per 28-day cycle. Two patients were dose reduced due to pre-existing renal impairment, one due to concern for potential cardiotoxicity.

Eleven of the 31 patients (35.5% of the total) met the criteria for MAHA at some point during their treatment. This included two patients that later developed severe TMA (6.5% of the total). There was a significantly higher mean age of affected vs unaffected patients (72 versus 64 years, *p* = 0.02), as seen in Table 1. The two groups had otherwise similar ethnic representation, myeloma immunophenotype and median number of prior lines of therapy.

**Table 1:** Patient baseline characteristics and survival—affected (patients who developed MAHA) vs unaffected (patients without MAHA).

	Affected (*n* = 11) 35.5%	Unaffected (*n* = 20) 64.5%
Mean age	72	64
Male	8	10
Female	3	10
Ethnicities	Caucasian/European (8) South East Asian (1) South Asian (1) South American (1)	Caucasian/European (13) South East Asian (1) South Asian (1) Middle Eastern (4) African (1)
Myeloma immunophenotype	IgG (6) IgA (4) Light chain (1)	IgG (10) IgA (2) IgD (1) Light chain (6) Non-secretory (1)
No. of prior treatment lines including autograft (no. of patients)	2 (2) 3 (4) 4 (1) 5 (2) 8 (1)	2 (2) 3 (8) 4 (4) 5 (3) 6 (1) 7 (2)
Median no. of prior lines	3	3.5
Median OS	21 months	21.5 months

*OS* overall survival from carfilzomib commencement.

All non-TMA episodes of MAHA were brief and low grade, with data detailed in Table 2. These episodes were associated with occasional schistocytes on peripheral film, compared with numerous schistocytes seen in both TMA cases. No patient had elevated bilirubin. There was a large variation in the onset of MAHA, ranging between cycle 2 and 19. Three of the 11 patients had concurrent infection during a MAHA episode. Five patients had more than one episode of MAHA. In one patient, carfilzomib was withheld for 2 weeks due to infection and peripheral oedema. In retrospect, this occurred during low-grade MAHA. In all other patients with low-grade MAHA episodes, carfilzomib was continued without immediate complications. The median overall survival from carfilzomib commencement (OS) was similar in both affected (21 months) and unaffected (21.5 months) groups.

**Table 2:** Affected patients and MAHA/TMA characteristics.

Patient no.	Age + sex	MAHA/TMA	Cycle onset no.	Schistocytes	Hb	Platelets	LDH	Retic	Haptoglobin Nadir	Bilirubin	Other	End organ damage	With infection	No. of episodes
15	84 M	MAHA	2	Yes	88	87	338	303	NA	12	-	Nil	-	1
16	65 M	MAHA	3	Yes	101	49	458	219	NA	10	-	Nil	-	1
21	56 M	**MAHA (subTMA)**	7	Yes	90	47	380	NA	NA	7	-	**AKI Cr 86–140**	Influenza A	1
29	64 F	MAHA	4	Yes	120	76	310	NA	NA	9	-	Nil	Rhinovirus	1
30	72 M	MAHA	4	Yes	94	89	269	245	Undetectable	12	Urine haemosiderin	Nil	-	2
36	81 F	**MAHA (subTMA)**	7	Yes	108	123	257	192	NA	4	-	**AKI Cr 176–225**	-	2
		TMA	12	Yes	66	120	313	164	Undetectable	7	Low C3, normal C4	Renal	-	
37	81 M	**MAHA (subTMA)**	3	Yes	89	72	255	NA	Low 0.2	8	-	**AKI Cr 111–138**	-	2
38	70 F	**MAHA (subTMA)**	5	Yes	92	66	417	NA	NA	4	-	**AKI Cr 90–117**	RSV	2
		TMA	17	Yes	92	27	987	12	Undetectable	11	-	Renal, Cardiac	Influenza A	
39	64 M	**MAHA (subTMA)**	6	Yes	81	68	415	NA	NA	14	-	**AKI Cr 119–216**	-	1
41	82 M	MAHA	6	Yes	94	100	507	208	NA	11	-	Nil	-	2
44	77 M	MAHA	7	Yes	101	58	344	91	Low 0.48	6	-	Nil	-	1

Units and normal ranges—Haemoglobin (Hb) males 130–170 g/L and females 120–150 g/L, Platelets 150–400 × 10^9^/L, LDH *<* 250 U/L, Reticulocyte count 50–100 × 10^9^/L, Haptoglobin 0.5–2.6 g/L, Bilirubin < 20 µmol/L, Creatinine (Cr) males 60–110 µmol/L, females 45–90 µmol/L.

*NA* not available, s*ub TMA* subclinical TMA (in bold).

Five of the 11 patients (45%) with low-grade MAHA had associated AKI, largely transient, except in one case. The increase in creatinine ranged between 27 and 80%, with a mean rise of 54.6%. These episodes of kidney injury had no other identifiable cause. AKI, together with MAHA, is indicative of previously unrecognised or subclinical TMA in these patients. There were no otherwise unexplained episodes of AKI in the unaffected group.

Both patients with overt TMA had severe manifestations. The first was 70 years old, with TMA occurring in the 16th cycle of carfilzomib during influenza A infection. ADAMTS-13 level was normal. Along with MAHA, she developed severe AKI with a creatinine peak of 530 µmol/L. Baseline creatinine was 100 µmol/L indicating mild chronic impairment, and interestingly, the renal biopsy revealed chronic TMA changes, including microvascular thrombi. She also developed moderate cardiac dysfunction, with a left ventricular ejection fraction of 35% and a normal coronary angiogram. Eculizumab was commenced on day 3 of the presentation and administered weekly for 4 weeks, then fortnightly for 6 months. MAHA resolved immediately; however, renal function showed initial early improvement, followed by gradual recovery over 3 months, with creatinine plateauing at approximately 200 µmol/L. With this plateau and echocardiogram confirming normalisation of cardiac function, eculizumab was ceased. At the next disease progression (PD), she was treated with lenalidomide and dexamethasone. She went on to have an OS of 46.5 months. The second patient with TMA was 81 years old and presented following her 12th cycle of carfilzomib. She had MAHA with severe anaemia, haemoglobin 66 g/L, as well as acute on chronic kidney injury, with creatinine rise from 256 to 404 µmol/L. Baseline creatinine with initial carfilzomib commencement was 138 µmol/L, with a gradual decline in renal function prior to acute TMA. ADAMTS-13 was not sent, given MAHA self-resolved with carfilzomib cessation. Unfortunately, renal function showed minimal recovery, despite a late trial of eculizumab. Due to persistent renal failure and frailty, she was palliated, with OS 15.1 months. In both TMA cases, carfilzomib was ceased immediately following presentation with severe AKI. Both patients had PD at approximately 3 months after cessation. The median OS in the remainder of the cohort was 21 months.

A recent analysis by Kozlowski et al. (2020) showed that haemolysis in carfilzomib-treated patients is indeed common, although often mild [10]. The rate of haemolysis reported in that study was somewhat higher, perhaps due to our use of strict multi-point criteria. However, in doing so, our study is the first to demonstrate that, in fact, this haemolysis is due to episodes of low-grade MAHA occurring in a large portion of carfilzomib-treated patients, and they are often unrecognised. We also found that older age is a significant risk factor for MAHA in carfilzomib therapy.

Our study showed the importance of recognising low-grade episodes of MAHA is twofold. First, almost half of these cases had associated AKI, indicating subclinical TMA. The rate of renal dysfunction (16%) in our study, not attributable to disease progression, is similar to that reported by Fotiou et al., where 17% of carfilzomib patients developed renal dysfunction, again often transient episodes. Among the patients without overt TMA, four of six that underwent renal biopsy showed evidence of acute and/or chronic TMA changes [6]. This is similar to our observation of chronic TMA changes on renal biopsy in one patient. Furthermore, we identified that this patient met the criteria for subclinical TMA earlier in the treatment course. Together this suggests that unrecognised subclinical TMA with repeated carfilzomib exposure can lead to chronic end organ damage through cumulative injury in susceptible patients. Additionally, in a portion of carfilzomib patients with unexplained renal dysfunction, although often low-grade, laboratory evidence of MAHA can be detected.

Second, both patients that developed severe TMA had evidence of a prior low-grade MAHA episode, and therefore this may be an indicator of increased risk in these patients. The two cases of severe TMA are a reminder that although uncommon, TMA remains a potential severe complication. In one case, prompt initiation of eculizumab resulted in rapid MAHA resolution and reversal of both renal and cardiac acute injury. The lack of renal recovery in the second case impacted survival and suggests the chronic injury is unlikely to improve, reinforcing the importance of early recognition of renal dysfunction and eculizumab initiation [2, 11].

Our results demonstrate that bilirubin is an insensitive marker in MAHA and even severe TMA. Studies have shown TMA can occur at any point during the treatment course [1, 2, 12, 13]. Our results indicate this is also true for low-grade MAHA, suggesting that in susceptible patients, a ‘complement amplifying’ stimulus such as infection may trigger episodes. In our study, concurrent infection was identified in three of the 11 patients.

Our analysis is limited by its retrospective nature and relatively small cohort. In isolation, several of the data points are not specific to haemolysis. Schistocyte reporting on peripheral film can be subjective when fragment numbers are low. Steps were taken to improve specificity by strict multi-point criteria for MAHA, as well as removing potential confounders with disease marker correlation. The above notwithstanding, this study is the first to demonstrate a spectrum of carfilzomib-induced MAHA, including low-grade episodes in a large portion of patients, subclinical TMA with kidney injury and, less commonly, overt TMA. Our study highlights the importance of monitoring patients receiving carfilzomib for haemolysis and end organ dysfunction, particularly in older patients and during concurrent infection. Given no significant difference in OS, our results indicate that upon detection of low-grade MAHA, carfilzomib can often be continued cautiously. However, this should prompt a review of benefit and risk, especially in the event of associated AKI. Ongoing work is required to improve the assessment of risk, such as complement gene mutations and management guidelines to prevent TMA.

## Data Availability

The de-identified total dataset can be provided on request to the corresponding author.
